# Factors associated with non-adherence to insulin in Type 1 and Type 2 diabetes mellitus patients in Western region of Algeria, Tlemcen: a cross-sectional study

**DOI:** 10.11604/pamj.2022.41.172.32972

**Published:** 2022-03-03

**Authors:** Mohammed Nassim Boukli Hacene, Yassamina Tabet Zatla, Meriem Saker, Zoubeyr Abbou, Amina Youcef, Houssam Boulenouar, Hakima Fekir, Khawla Khaldi, Nawel Brikci-Nigassa, Ali Lounici, Kaouel Meguenni

**Affiliations:** 1 Department of Pharmacy, Faculty of Medicine, University Abou-Bekr Belkaïd, Tlemcen 13000, Algeria,; 2 Department of Biochemistry, Dr Tidjani Damerdji University Hospital, Tlemcen, Algeria,; 3 Cancer Research Laboratory n°30, Abou-Bekr Belkaïd University, Tlemcen, Algeria,; 4 Department of Mathematics, Faculty of Medicine, University Abou-Bekr Belkaïd, Tlemcen 13000, Algeria,; 5 Manchester University NHS Foundation Trust, Manchester, United Kingdom,; 6 Laboratory of Physiology, Pathophysiology and Biochemistry of Nutrition, Department of Biology, Faculty of Natural and Life Sciences, Earth and Universe, University Abou-Bekr Belkaïd, Tlemcen 13000, Algeria,; 7 Department of Internal Medicine, Diabetes Research Laboratory, Faculty of Medicine, University Abou-Bekr Belkaïd, Tlemcen 13000, Algeria,; 8 Department of Epidemiology, Dr Tidjani Damerdji University Hospital, Abou-Bekr Belkaïd University, Tlemcen, Algeria

**Keywords:** Adherence, diabetes mellitus, insulin therapy, Algeria

## Abstract

**Introduction:**

adherence to drugs is critical for achieving the best clinical results in the treatment of chronic diseases. Adherence to chronic drugs might be influenced by beliefs about medications and other variables. The goal of this study was to assess relevant determinants of medication adherence in Algerian population with insulin-dependent diabetes.

**Methods:**

from July 1^st^ 2019 to February 29^th^ 2020, a cross-sectional study was conducted. Participants who had been on insulin for at least 6 months were recruited from Tlemcen (Algeria) clinics in secondary care settings. Patients were invited to a face-to-face interview, in order to complete out the Morisky Medication Adherence Scale-8 (MMAS) tools to report their attitudes towards medication adherence and views about their insulin. The recruited patients' socio-demographic data was also collected. The related determinants of chronic drug non-adherence in the tested population were identified using a stepwise binary logistical regression model.

**Results:**

in this study, 147 patients out of 400 were not adhering to their insulin therapy (36.5%). Insulin non-adherence was linked to single status (AOR=2.088, CI=1.180-3.694), non-insurance (AOR=2.949, CI=1.323-6.572), number of daily insulin injections (AOR=1.269, CI=1.033-1.559), unawareness of the insulin regimen (AOR=3.528, CI=1.453-8.565), hypertension (AOR= 3.497, CI=1.98-6.154) and the non-practice of self-monitoring of blood glucose (SMBG) (AOR=2.635, CI=1.472-4.718).

**Conclusion:**

insulin adherence in Algerians is still well below international standards. This study improved the understanding of the factors affecting the non-adherence to insulin among diabetics and may be used as a baseline to target; throughout educational programs; the sub-populations identified as non-adherents.

## Introduction

According to the International Diabetes Federation´s latest figures, the incidence of diabetes in Algeria has increased to 7.2% of people aged from 20 to 79, or one adult every 16 people. Algeria is one of the top ten countries in the world for both the number of children with type 1 diabetes and the number of new cases of type 1 diabetes [1]. As a result, improving diabetes management remains a national public health priority.

Insulin therapy is the gold standard for treating type 1 diabetes mellitus which is caused by a complete lack of this hormone. It´s uncommonly used in the early stages of type 2 diabetes but frequently as a last resort once the condition has progressed in this population [2]. Insulin initiation difficulties are a key barrier to diabetic patient treatment at its best. When physicians talk to their patients about this, they often hear concerns about hypoglycemia, discomfort, and weight gain, as well as a refusal to use a restricted drug that does not allow for injection schedule flexibility. White coats, too, put off this critical time, particularly when they are aware of their inability to give enough insulin injection training and comprehension to ensure patients´ adherence [3].

The terms “compliance” and “adherence” are frequently used interchangeably. Compliance refers to a patient´s willingness to take the drugs given by a doctor, and it implies that the patient is blindly following the doctor's advice. Therapeutic adherence is distinct in that it respects (takes into account) the patient´s viewpoint and entails collaboration between the care provider and the patient in achieving and implementing the therapeutic care plan. As a result, “adherence” is being increasingly widely used in medical, biological, and health-related research [4]. Despite the fact that therapeutic adherence has been thoroughly described in the literature, Becker and Maiman´s quote from 35 years ago, “Therapeutic adherence has become the best known but least understood health behavior”, and it remains important and summarizes the current state of our understanding in this field [5].

Improving medication adherence may have a greater influence on the health of our population than in the discovery of any new therapy [6], a quote that is both meaningful and relentlessly real. Regarding diabetes, non-adherence has been recognized for some time as increasing the risk of mortality [7]. Also, specific non-adherence to insulin has been shown to impair glycemic control, lead to higher hospitalization rate [8] and recently to increase mortality [9]. Moreover, insulin is one of the most effective glucose-lowering agents [10] and the fact that patients may opt to not take it, intentionally or unintentionally, is questionable [11]. Therefore, the evaluation of insulin adherence and the identification of the reasons for non-adherence are major issues in the management of patients on insulin. Since these parameters are still insufficiently studied in the Algerian population, this will be the first research aimed at evaluating insulin adherence in Algerian patients and examining the factors that influence it.

## Methods

### Study setting and subject

This descriptive cross-sectional study was conducted over a period of 08 months, from July 1^st^ 2019 to February 29^th^ 2020. All patients who visited the internal medicine department of Tlemcen´s University Hospital Center, and those attending the outpatient clinics of Agadir and Boudghen within the Tlemcen´s urban area, were invited to participate in the study if they met the following criteria: 1) diagnosed with either type 1 or Type 2 diabetes, 2) being on insulin since at least 6 months. Patients were excluded from the study if they were pregnant, naïve on insulin or with severe mental illness.

The study was conducted as per the Declaration of Helsinki and Good Clinical Practices and approved by the Institutional Review Board of the Department of Pharmacy. Eligible patients were informed of the goals and methodology, as well as the confidentiality of the data collected and the fact that they could either consent or refuse to participate. They would be provided with a formal consent form if they agreed to participate, in which they would agree to complete questionnaires, provide a blood sample, and/or have access to their blood results. To calculate the sample size, the average percentage of non-adherence of 31.3% noted in a recent Algerian study carried out on type 2 diabetics were taken into account [12]. With a confidence level of 95% and an error margin of 5%, a total of 316 patients was needed to conduct the study.

### Data collection

To collect socio-demographic and clinical information about diabetes and its complications, researchers performed face-to-face interviews with patients who self-inject insulin or family who deliver regular injections. The internal medicine department's employees collected blood samples in a methodical manner for biological examination at the Tlemcen's hospital's biochemistry department. After giving their agreement, patients treated (on an outpatient basis) in Agadir and Boudghen were referred to the Tlemcen's Hospital's biochemistry department for sampling. The measures studied were fasting blood glucose, Hemoglobin A1C (HbA1c), total cholesterol, TG, and serum creatinine. Finally, we went through the patient's files at the Internal Medicine Department, obtained the results from the Biochemistry Department, or called the individuals directly to obtain their biological results.

### Operational definition

The weight was divided by height squared to determine the body mass index (Kg/m^2^), which was then graded according to WHO guidelines [13]. HbA1c glycemic control was considered good when was<7% and poor when was ≥7%. When the fasting blood glucose target was less than 1.26 g/L, it was considered as achieved, and when it was more than 1.26 g/L, it was considered as poor and not meeting the target [14]. According to the American Diabetes Association, cholesterol and triglyceride levels are regulated when blood concentrations are less than 2 g/L and 1.50 g/L, respectively [15]. The MDRD (Modification of Diet in Renal Disease) formula was used to measure the glomerular filtration rate, which is adjusted for a body surface area of 1.73 m^2^ and does not include the patient's weight [16]. The stages of chronic renal failure based on the value of glomerular filtration rate (ml/min/1.73m^2^) have been categorized as Stage 1 ≥ 90, Stage 2: 60-89, Stage 3: 30-59, Stage 4: 15-29 and Stage 5 < 15 [17].

### Measuring insulin adherence (Morisky scale)

The Morisky Medication Adherence Scale-8-item was used to test insulin adherence (MMAS-8). The questionnaire was given in French or Arabic, but we often translated the questions into Algerian accent to ensure that we were understood. Non-adherence is indicated by a total score of less than 6, while adherence is indicated by a total score of 6 or more on the MMAS-8.

### Statistical analysis

A descriptive analysis was performed to describe the socio-demographic and therapeutic characteristics, the underlying conditions and the biological profile of the studied population. The participants in the study were divided into two groups: adherents and non-adherents. The Chi-2 square test was used in the nonparametric study to examine the correlations between the dependent variable “Non-adherence” and all the variables that potentially affect it or the exact Ficher test when the Chi-2 test´s reliability criteria were not met. A value of p 0.05 was used to determine statistical significance. For the calculation of the odds ratio (OR) for dichotomous independent variables, 2x2 tables with confidence intervals were generated (IC95%). A binary stepwise logistic regression was used to predict the dichotomous variable insulin adherence. Patients were divided into two groups: adherent and non-adherent. Factors having a P value less than 0.10 in the uni-variate analysis were submitted to a multiple predictor analysis using the binary stepwise logistic regression approach in order to develop a model with variables that better-predicted insulin non-adherence in the study population. The odds ratio was used to determine the relative impact of each independent variable on the dependent variable “insulin non-adherence”.

### Data availability

The datasets used and/or analyzed during the current study are available from the corresponding author on reasonable request.

## Results

There were a total of 400 patients, including 135 men and 265 women, for a sex ratio of 1: 2. The patients ranged in age from 5 to 95 years old. The average age of the patients was 54.46 (±17.67) years. The fifties and sixties were the well-represented age groups, with 105 and 106 patients per class, respectively. 66.25% of those surveyed were overweight or obese, 31.25% were single, divorced, or widowed, 38.25% had never attended school, and 80% were unemployed. Almost all of the patients were on modest to medium incomes, and 10.25% did not have health insurance.

Type 1 diabetic´s account for 21.25% of the patients recruited, while type 2 diabetics account for 78.75%. In the study, population diabetes had been present for an average of 13.55 (± 8.293) years. There were almost as many diabetics on insulin alone (52.75%) as on Insulin and ADO (47.25%). The average age of onset of diabetes was 13.55 ± 8.292) years, the average age of diagnosis was 41.11 (± 16.412) years and the average time on insulin was 7.66 (± 6.704) years. Only 23% of our population was on a single insulin injection per day schedule, while 39% were on a four-insulin-injection-per-day diet. In our sample, 74.75% of the 400 patients had HbA1c ≥ 7% levels that were either above or below the guideline limits (mean HbA1c). The average fasting blood glucose level was 1.91 g/L, and approximately three out of four patients did not reach the targeted fasting blood glucose (FBG) level. For total cholesterol, 32.75% were unable to remain within prescribed limits, and as for triglycerides, this figure increased to 41%.

### Predictors/factors associated with non-adherence to insulin

In the survey participants, 254 patients (63.5%) were insulin adherent, while 147 patients were non-adherent (36.5%). Table 1 summarizes insulin therapy adherence as a feature of the sample population's and their socio-demographic characteristics. With p-values of 0.000 and 0.002, social status and social coverage were found to be linked to adherence.

**Table 1:** single predictor analysis of sociodemographic parameters associated with insulin non-adherence

Variable		Total	N of Non-Adherents (%)	P	OR	CI (95%)
**Gender**				0.705	1.086	0.707-1.669
	Male	135	51 (37.8%)			
	Female	265	84 (35.8%)			
**Age (y), mean (S.D.) =54.46 (17.672)**				0.068		
	<40	78	36 (46.1%)			
	40-59	137	52 (37.9%)			
	≥60	185	58 (31.3%)			
**BMI (Kg/m^2^) mean (S.D.) =27.46 (5.489)**				0.14		
	Underweight	12	5 (41.7%)			
	Normal	122	52 (42.6%)			
	Overweight	146	43 (29.5%)			
	Obese/Morbid Obese	119	46 (40.9%)			
**Marital status**				0.000*		
	Married	275	84 (30.5%)			
	Single	64	37 (57.8%)			
	Divorced, or separated	61	25 (40.98)			
**Education level**				0.267		
	Never attended school	153	49 (32.0%)			
	Primary	86	38 (44.2%)			
	Middle	74	28 (37.8%)			
	Secondary	54	22 (40.7%)			
	High school	33	9 (27.3%)			
**Occupation**				0.582		
	Public	52	18 (34.6%)			
	Private	27	9 (33.6%)			
	Self boss	1	1 (100%)			
	None	320	118 (36.9%)			
**Salary level**				0.896		
	Bottom	61	23 (37.7%)			
	Medium	329	120 (36.5%)			
	High	10	3 (30.0%)			
**Insurance coverage**				0.002*	2.743	1.419-5.299
	No	41	24 (58.5%)			
	Yes	359	122 (34.0%)			

P-value: * Significant at 0.05 level. OR: Odds ratio. CI: Confidence interval

Among the clinical parameters, type of diabetes, number of daily insulin injections, ability to describe the insulin regimen, frequency of self-measurement of blood glucose levels and hypertension all showed statistical significance toward insulin adherence in univariate research (Table 2). HbA1c, FBG, triglycerides, and glomerular filtration rate (GFR) were the four biologic parameters that affected insulin adherence (Table 3). In the univariate analysis, the variables that were statistically correlated with non-adherence were incorporated into the multivariate analysis model (Table 4). After adjustment, the nine factors found were independently, individually and correlated with insulin non-adherence, including single status (AOR: 2.088, p-value: 0.011), number of daily insulin injections (AOR: 1.269; p-value: 0.023), inability to describe insulin regimen (AOR: 3.528, p-value: 0.005), presence of hypertension (AOR: 3.497, p-value: 0.000), inability to control fasting blood glucose (AOR: 2.716, p-value: 0.003), inability to control triglycerides (AOR: 2.522, p-value: 0.000), glomerular filtration rate (AOR: 1.794, p-value: 0.002), non-practice of SMBG (AOR: 2.635, p-value: 0.001) and being uninsured (AOR: 2.949, p-value: 0.008).

**Table 2:** single predictor analysis of clinical parameters associated with insulin non-adherence

Variables		Total	N of Non-Adherents (%)	P	OR	CI (95%)
**Type of Diabetes**				0.011*	1.864	1.147-3.029
	Type 1 Diabetes	85	41 (48.2%)			
	Type 2 Diabetes	315	105 (33.3%)			
**Duration of Diabetes, mean (S.D.)= 13.55 (8.293)**				0.305		
	1	72	23 (31.9%)			
	2	104	45 (43.3%)			
	3	82	32 (39.0%)			
	4	72	26 (36.1%)			
	5	70	20 (28.6%)			
**Age at diagnosis (y), mean (S.D.)= 41.11 (16.412)**				0.18		
	1-10	24	10 (41.7%)			
	11-20	36	20 (55.5%)			
	21-30	29	12 (41.4%)			
	31-40	80	27 (33.8%)			
	41-50	114	39 (34.2%)			
	51-60	71	26 (36.6%)			
	61-70	46	12 (26.1%)			
**Years on insulin, mean (S.D.)= 7.66 (6.704)**				0.65		
	1-5	206	68 (33.0%)			
	6-10	97	39 (40.2%)			
	11-15	43	18 (41.9%)			
	16-20	30	11 (36.7%)			
	>20	24	30 (41.7%)			
**Current regimen**				0.213	1.297	0.861-1.953
	Insulin	211	83 (39.3%)			
	Insulin +OAD	189	63 (33.3%)			
**Number of daily insulin injections, mean (S.D.)= 3.02 (1.324)**				0.002*		
	1	92	27 (29.3%)			
	2	37	11 (29.7%)			
	3	78	18 (23.1%)			
	4	159	75 (47.2%)			
	5	34	15 (44.1%)			
**Ability to describe insulin regimen**				0.000*	3.467	1.698-7.079
	No	36	23 (63.9%)			
	Yes	364	123 (33.8%)			
**Practice of SMBG**				0.000*		
	No	86	47 (55.7%)			
	Yes	314	99 (31.5%)			
**Hypertension**				0.001*	0.486	0.320-0.727
	No	193	54 (28.0%)			
	Yes	207	92 (44.4%)			
**Dyslipidemia**				0.061	1.493	0.980-2.274
	No	236	95 (40.3%)			
	Yes	164	51 (31.1%)			

P-value: * Significant at 0.05 level. OR: Odds ratio. CI: Confidence interval

**Table 3:** single predictor analysis of biologic parameters associated with insulin non-adherence

Variable		Total	Nof Non-Adherents (%)	P	OR	CI (95%)
**HbA1c (%), mean (S.D.) =8.33 (1.925)**				0.001*	0.422	0.251-0.709
	<7%	101	23 (22.8%)			
	≥7%	299	123 (41.1%)			
**FBG (g/L), mean (S.D.) =1.93 (1.004)**				0.000*	0.359	0.211-0.611
	<1.26 g/L	102	21 (20.6%)			
	≥1.26 g/L	298	125 (41.9%)			
**Total Cholesterol (g/L), mean (S.D.) =1.90 (1.149)**				0.596	0.89	0.577-1.371
	< 2	268	95 (35.4%)			
	≥ 2	131	50 (38.2%)			
**Triglycerides (g/L), mean (S.D.) =1.45 (0.705)**				0.000*	0.467	0.308-0.707
	<1.50	236	69 (29.2%)			
	≥1.50	164	77 (47.0%)			
**GFR (ml/min/1.73m^3^), mean (S.D.) =74.97 (29.84)**				0.014*		
	Stage 1≥ 90	88	46 (52.3%)			
	Stage 2 : 60-89	188	59 (31.4%)			
	Stage 3 : 30-59	110	38 (34.5%)			
	Stage 4 : 15-29	9	2 (22.2%)			
	Stage 5 < 15	3	1 (33.3%)			

P-value: * Significant at 0.05 level. OR:Odds ratio. CI: Confidence interval

**Table 4:** logistic regression of factors associated with insulin non-adherence

	B	S.E	P-Value	AOR	CI (95%)
**Single status**	0.736	0.291	0.011*	2.088	1.180-3.694
**Number of daily insulin injections**	0.239	0.105	0.023*	1.269	1.033-1.559
**Inability of describing insulin regimen**	1.261	0.453	0.005*	3.528	1.453-8.565
**Hypertension**	1.252	0.288	0.000**	3.497	1.987- 6.154
**Non-control of FBG**	0.999	0.340	0.003**	2.716	1.394-5.291
**Non-control of Triglycerides**	0.925	0.252	0.000**	2.522	1.539-4.131
**Glomerular Filtration Rate**	0.584	0.186	0.002**	1.794	1.246-2.581
**Non-practice of SMBG**	0.969	0.297	0.001**	2.635	1.472-4.718
**Unassured**	1.081	0.409	0.008**	2.949	1.323-6.572

Odds ratios adjusted according to the following variables: social status, insurance coverage, type of diabetes, number of daily insulin injections, ability to describe insulin regimen, self-monitoring blood glucose, hypertension, controlling HbA1c, controlling FBG, Controlling Triglycerides, glomerular filtration rate. P-value from the multiple linear regression: * Significant at 0.05 level ** Significant at 0.01 level B: The coefficient of the constant in the null model, S.E: Standard error around the coefficient of the constant in the null model, AOR: Adjusted odds ratio. CI: Confidence interval.

## Discussion

According to the 8-item Morisky questionnaire, 36.5% were not insulin adherent. Scores similar to 31.3% in an Algerian study on type 2 diabetics (on ADO and/or insulin) [12], 33.1% in an Ethiopian study of a similar size population [18], but lower than 88.1% in a Pakistani study of type 1 diabetics on insulin [19] were published. This lack of insulin adherence may be unintentional or intentional. When forgetfulness is unintentional, it is often highlighted as a product of occupation or distraction [20]. Intentional insulin non-adherence is also concerning, particularly when 50% of participants in an American study reported being adept of it and 20% (among them) on a regular basis [21]. The average lack of adherence to insulin exceeded 3.3 days/month in another study by the same U.S. team, which was performed on a larger population [22]. Among the socio-demographic characteristics evaluated, only social position and insurance coverage were linked to insulin non-adherence. Living alone (due to non-marriage, spouse death, or divorce) increased the chance of insulin medication non-adherence by double (AOR: 2.088; p-value: 0.011). Single patients' low insulin adherence could be the result of a “marriage crisis” that has afflicted Algerians for decades [23]. The diabetic's partner (the person who lives with him or her on a daily basis) may be able to provide the emotional and psychosocial support needed to improve commitment and insulin adherence [24,25].

In this study, non-insurance coverage tripled the likelihood of insulin non-adherence AOR: 2.949; p-value: 0.008). It´s worth noting that Algerian insurance companies cover “all anti-diabetic drugs - oral anti-diabetic drug (OAD) and insulin”. Because a box of insulin costs one-third of the guaranteed minimum income, being under social security (insured) becomes a luxury. This could explain why, in the absence of insurance, adherence is so weak. However, this is only the visible aspect; in fact, the health system, which is at the end of the chain, bears the brunt of it the most. Indeed, Chandran *et al*. (2015) found that if a patient sticks to his insulin regimen, the cost curve inverts, resulting in thousands of dollars in annual savings [26]. Ayyagari *et al*. (2015), reached the same conclusion in terms of medication, but they outbid the medical factor by stating that good insulin pen adhesion greatly decreases hospitalizations and their high cost to the health-care system [8]. In a previous publication, we recommended that Algerian insurance providers pay for insulin pen needles [27], and we reinforce the same recommendations in order to find a solution for uninsured diabetics on insulin.

One would expect that since insulin is the cornerstone of type 1 diabetes care, type 1 diabetics will be more hesitant to give up its intake in comparison to type 2 diabetics, especially those on OAD, for whom insulin is less important. The multicenter study by Peyrot *et al*. (2012) on approximately 1,530 diabetics on insulin [22] and the study by Farsaei *et al*. (2014) on 507 patients both support this pattern [28]. Our findings show that non-adherence to insulin is more significant in Algerian type 1 diabetics than in type 2 diabetics, which defies logic (48.2% VS 33.3%). According to a recent Algerian survey, 69.1% of type 1 diabetics obtained insulin education, but the degree of that education was not mentioned [29]. Particular attention is required for these diabetics through therapeutic education, focusing on the key issue of adherence and the fatal risk of medication omission. In several studies, the basal-bolus system, which involves one or two basal insulin injections and multiple bolus insulin injections based on the number of daily meals and physical activity, has been linked to a higher risk of dose skipping and, as a result, non-adherence [20,30]. Our findings support this theory, as patients who inject insulin five times a day are the least adherent in our population.

Glycemic control is crucial for diabetic patients, particularly those on insulin, since it helps prevent diabetes-related complications. Although the optimum frequency is not well described in the literature, the Algerian insurance scheme covers the reimbursement of many glycemic strips a day for its insulin-dependent beneficiaries. Patients in our survey don't dispute it: 78.5% test regularly, which matches the Algerian findings (73.1%) from Wave 7 of the International Diabetes Management Practices Study.

Regular capillary blood glucose regulation contributes to better insulin adherence in this study. Patients who did not monitor capillary blood glucose levels were 2.635 times more likely to be insulin non-adherent than those who did (p-value: 0.001). This may be explained by the fact that repeated SMBG can provide constructive feedback on the efficacy of the adherence, motivating the patient to do more rigorous self-monitoring. Just 62% of 403 diabetic patients in an Algerian study followed the guidelines for good glycemic self-monitoring practice [29]. For an optimal control, it would be better to combine quantity (frequent monitoring) and consistency (optimal SMBG) (adherence to recommendations).

Some patients in our research were unable to correctly identify their insulin regimen and, as a result, had weaker adherence. Since almost all insulin patients self-inject, this is understandable. Adherence is identified as a critical factor in reducing diabetes' long-term complications [31]. Any comorbidities can have an effect on it. Hypertension was reported as the most common risk factor, with a prevalence of 32.7%, in a study of cardiovascular risk factors in the Tlemcen area, the site of our study. Another Algerian multicenter study of over 977 diabetics found that 55.5% of them had hypertension [32]. Hypertension, which affects 48.25% of the surveyed population, was highlighted to be the only diabetes-related complication linked to non-adherence in this study (risk increased by a factor of 3.497, p-value: 0.000). The poor adherence rate among these patients may be explained by the need to assimilate a repulsive complex insulin regimen with additional, possibly multiple regular doses of antihypertensive medications [3,4,33,34].

The highest predictor of good glycemic regulation is HbA1c. In this study, 74.8% of insulin patients failed to lower this parameter below the ADA´s (American Diabetes Association) recommended limit of 7%, compared to 64.6% in a large-scale Algerian survey of more than 12390 diabetic patients, 66.33% in Moroccan type 2 diabetics, and 85% in a Sudanese population of the same type [35-37]. Most studies examining adherence in diabetic patients attempt to link it to this endpoint in this way. Patients with HbA1c levels above appropriate (ones) were found to be less adherent, in this current report. According to some studies, when a diabetic patient sticks to his or her care plan, his or her glycemic condition increases [31,38-40]. Despite the fact that it is not as reliable because subjective and measured in one shot, fasting blood glucose was found to be higher in adherent insulin patients.

The Algerian “A1chieve study”, which included 1,494 diabetics, found that starting basal insulin improved glycemic (HbA1c, FBG, postprandial glucose (PPG)), lipid (total cholesterol (TC) and triglyceride (TG), and quality of life parameters after 24 weeks without causing hypoglycemic problems or substantial weight gain [41]. The Algerian “ADHERE research”, which included nearly 575 type 2 diabetic patients who were not controlled on OAD and were starting on basal insulin, found that 84.2% had good adherence after 12 months of treatment, particularly those who had received therapeutic education (a positive predictive factor of adherence) [42].

There was no correlation between age and poor insulin adherence in this research. In chronically debilitated patients, age is frequently identified as a major determinant of poor therapeutic adherence, especially due to poly-medication and cognitive disorders [43]. In a study by Egede *et al*. (2011) [38], it was discovered to be a negative predictor of insulin adherence, although other studies were unable to show a connection [20,21,43,44]. In this research, obesity had no effect on adherence, despite a publication claiming that patients skip insulin injections in an attempt to lose weight [44].

Other determinants influencing insulin therapeutic adherence have been identified in the literature such as quality of life, understanding of the risk of hypoglycemia, and changing injection material [31,45,46]. There are also some beliefs listed. Alyami *et al*. (2019) investigated the impact of beliefs on diabetic adherence, especially the role of the Good Lord as a health-status regulator. Patient non-adherence was linked to the belief that health status (implied diabetes) is influenced by God's will in the Saudi Muslim society where the survey was conducted [47].

### Limitations and strengths of the study

The following constraints are acknowledged. Firstly, there was no randomization; only patients who gave their consent and met the inclusion criteria were considered. Also, biological results were obtained at Time T with no subsequent monitoring or previous records. Therefore, patients seen in the Tlemcen area´s free public hospital and/or clinics were excluded from the investigation. As a result, the findings cannot be applied to the entire population. Moreover, patients´ replies that rely on “subjective” recall, which could lead to patients lying to avoid being judged adversely, could impact Morisky´s questionnaire, which has been validated as a measure for monitoring clinical adherence. Finally, some non-adherence factors mentioned in the literature may not have been investigated. This study is not lacking in content. For the first time, factors impacting insulin adherence among Algerian patients were explored. The findings are likely to be reflective of insulin users in the Tlemcen area. Interestingly, instead of phone interviews or self-administered questionnaires, face-to-face interviews were useful in explaining some of the form questions and information that a patient might misunderstand. This strategy had a missed answer rate of roughly 2%, which is insignificant given the amount of data collected. Lastly, the association between clinical adherence and HbA1c, a key marker of diabetic control, was investigated in this study.

## Conclusion

As far as we know, this is the first survey in Algeria that measured insulin therapeutic adherence and gave data on the determinants of insulin non-adherence. Insulin adherence was found to be low in patients from the Tlemcen area. Several Subpopulations non-adherent to their insulin regimen were identified. These should be targeted by therapeutic educational programs.

### What is known about this topic

- *Insulin is used in the two main populations of diabetics, type 1 and type 2;*
- *Adherence to insulin is critical for achieving glycemic goals*.

### What this study adds

- *Adherence to insulin was estimated for the first time in Algerian type 1 and type 2 diabetics;*
- *Insufficient adherence to insulin was retrieved as more than one third of the study population was non-adherent;*
- *Key predictors of insulin non-adherence were identified including single status, insurance un-coverage and the non-practice of self-monitoring blood glucose*.
